# The MOTIV-HEART Study: A Prospective, Randomized, Single-Blind Pilot Study of Brief Strategic Therapy and Motivational Interviewing among Cardiac Rehabilitation Patients

**DOI:** 10.3389/fpsyg.2017.00083

**Published:** 2017-02-07

**Authors:** Giada Pietrabissa, Gian Mauro Manzoni, Alessandro Rossi, Gianluca Castelnuovo

**Affiliations:** ^1^Psychology Research Laboratory, Ospedale San Giuseppe, IRCSS Istituto Auxologico Italiano IRCCSVerbania, Italy; ^2^Department of Psychology, Catholic University of MilanMilan, Italy; ^3^Faculty of Psychology, eCampus UniversityNovedrate, Italy

**Keywords:** cardiovascular diseases, brief strategic therapy, motivational interviewing, cardiac rehabilitation, pilot study

## Abstract

**Background:** Psychological distress, biomedical parameters, and unhealthy lifestyles contribute to a poorer prognosis for cardiac disease. Public health's challenge is to motivate patients to utilize self-care.

**Objective:** This prospective, randomized, single-blind pilot study aimed at testing the incremental efficacy of Brief Strategic Therapy (BST) combined with Motivational Interviewing (MI) in improving selected biomedical and psychological outcomes over and beyond those of the stand-alone BST in a residential Cardiac Rehabilitation (CR) program.

**Method:** Fourty-two inpatients (17 females), enrolled in a 1-month CR program, were randomly allocated into two conditions: (a) Three sessions of BST and (b) Three sessions of BST plus MI. Data were collected at baseline, discharge, and after 3 months through phone interviews.

**Results:** At discharge, no significant between-group difference was found in any outcome variable. Changes from pre- to post-treatment within each condition showed significant improvements only in the BST group, where the level of external regulation diminished, and both the participants' self-regulation (Relative Autonomous Motivation Index, RAI) and willingness to change improved. At the 3-month follow-up, within-group analyses on responders (BST = 9; BST + MI = 11) showed a statistically significant improvement in the level of systolic blood pressure in both groups.

**Discussion:** Findings showed no evidence of the incremental efficacy of combining BST and MI over and beyond BST alone on either selected biomedical or psychological outcomes among CR patients.

**Conclusions:** Ends and limitations from the present pilot study should be considered and addressed in future investigations.

## Introduction

Cardiovascular Disease (CVD) is a global health issue and leading cause of morbidity and mortality in developed countries (Beauchamp et al., 2010).

Psychological factors, such as lack of social support, depression, anxiety, and type A behavior/hostility, largely contribute to the etiopathogenesis of heart disease (Rozanski et al., 1999; Favoccia et al., 2014). They may also impact a wide range of health, functioning, and Health-Related Quality of Life (HRQoL) outcomes for persons with CVD (Macleod and Davey Smith, 2003). Psychosocial determinants may, in fact, cause direct acute or chronic pathophysiological changes (Hemingway and Marmot, 1999). They may also affect adherence to treatment and long-term maintenance of health related behaviors (e.g., smoking, diet, alcohol consumption, or physical activity), which in turn increase the risk of developing CVD (Ceccarini et al., 2015; Pietrabissa et al., 2015b). Particularly, lifestyle choices represent central barriers in differentiating cardiac diseases (Cole et al., 2011), since they are highly related to the development of other chronic conditions, such as obesity (Ignarro et al., 2007; Yang et al., 2011; Castelnuovo et al., 2015a).

Obesity is an independent predictor of CVD (Hubert et al., 1983) through its influence on hypertension, hypercholesterolemia, type 2 diabetes, left ventricular hypertrophy, and individuals' Functional Capacity (FC; Pietrabissa et al., 2012; Castelnuovo et al., 2013) and, at entry into Cardiac Rehabilitation (CR), the majority of patients are generally overweight or obese (Gunstad et al., 2007). Moreover, obesity presents both direct and indirect costs, respectively, resulting from treatment of morbidity and productivity loss, with augmented individual, national, and global healthcare expenditures (Castelnuovo et al., 2015b).

CR programs are essential in improving the well-being of CVD patients through the delivery of customized plans for exercise training, education on heart-healthy living, and counseling to reduce stress (Pack et al., 2013). Notably, in the up-to-date practice of CR, psychosocial services have grown in importance and role (Greco et al., 2011), and new psycho-educational interventions are promoted as a means to improve outcomes in CR (Humphrey et al., 2014; Ginsberg et al., 2015).

In this regard, the Brief Strategic model of Therapy (BST) has recently achieved varying degrees of success in eliminating dysfunctional symptoms or behaviors by producing changes in patients' perceptions of and reactions to their personal and interpersonal reality (Nardone and Portelli, 2005; Castelnuovo et al., 2011; Pietrabissa et al., 2016). The strategic therapist is not interested in discovering why a certain problem exists, but in what is maintaining it in the present, so as to interrupt a given vicious circle through the flexible use of rigorous, but not rigid, treatment protocols (Nardone and Watzlawick, 2005) and communication techniques (*strategic dialogue*; Nardone and Salvini, 2007; Pietrabissa et al., 2015b). Still, since the ability to engage in self-management behaviors is particularly important to prevent additional complications among patients with a cardiac problems (Hagger, 2010), alternative-integrative strategies are needed that specifically focus on raising both the individuals' readiness to change (Kreman et al., 2006; Pietrabissa et al., 2015a) and self-efficacy (Burke et al., 1997; Miller et al., 1997; Pietrabissa et al., 2013). To this aim, the American Heart Association recommends Motivational Interviewing (MI) as an effective approach for promoting behavioral change (Artinian et al., 2010). MI is a non-judgmental, guiding communication style that works to enrich people's competence and autonomy, as well as to facilitate and engage their intrinsic motivation in order to elicit (long-lasting) behavioral change (Miller and Rollnick, 2013). Individuals, in fact, usually know what they should or should not do for their health but generally fail to take action. Therefore, a patient-centered approach to consultations, focused on exploring and resolving the ambivalence regarding change, is usually preferred to a more directive advice giving technique (Little et al., 2001). Evidence exists for the efficacy of MI in increasing physical activity (Brodie and Inoue, 2005; Thompson et al., 2011), reduced caloric intake (Carels et al., 2007; Martins and McNeil, 2009) and decreased Body Mass Index (BMI; Woollard et al., 2003; Hardcastle et al., 2013) among patients with CVD. Despite its largely atheoretical origins, Self-Determination Theory (SDT) recently has been used as a de facto model for understanding how and why MI works (Vansteenkiste and Sheldon, 2006). Originally proposed by Ryan and Deci (2000), SDT suggests a multidimensional conceptualization of motivation in which the different regulations are said to fall along a continuum of self-determination, ranging from completely external (therefore not internalized), to being regulated by internal pressures, to being completely self-regulated. MI can be used in conjunction with other forms of psychotherapy (Macgowan and Engle, 2010; Dietz and Dunn, 2014) at different exposure times (Palacio et al., 2016) and can be well-integrated into brief patient encounters (Rubak et al., 2005). In order to modernize CR services, it is essential to understand how multiple psychological and biological factors interact in the regulation of the cardiovascular system and the development of CVD, as well to design effective interventions able to manage psychological impairments and capture the range of mechanisms involved in the behavioral change process.

To this aim, the present pilot study, MOTIV-HEART (MOTIVational strategies for patients with HEART disease), contributes to a gap in the literature by testing the incremental efficacy of BST within a residential 1-month CR program and at a 3-month follow-up, including motivational components that improve/maintain selected biomedical and psychosocial outcomes over and beyond a stand-alone BST (the routine psychological practice restricted to the CR Unit where the present investigation was conducted).

The primary hypotheses are that patients assigned to the experimental condition, compared to those receiving BST only, will show: (i) higher level of self-regulation (Relative Autonomous Motivation Index, RAI) at discharge from CR, and (ii) greater reductions in Kilograms (Kg) at 3 months follow-up. The secondary hypotheses are that (i) improvements in the level of anxiety, depression, impulsiveness, readiness to change, self-regulation, perceived self-efficacy and Health-Related Quality of Life (HRQoL), as well as in (ii) Low-Density Lipoprotein Cholesterol (LDL), Systolic Blood Pressure (SBP) and glucose level, will be maintained or further increased at the 3-month follow-up.

## Methods

### Study participants

**Figure 1:**
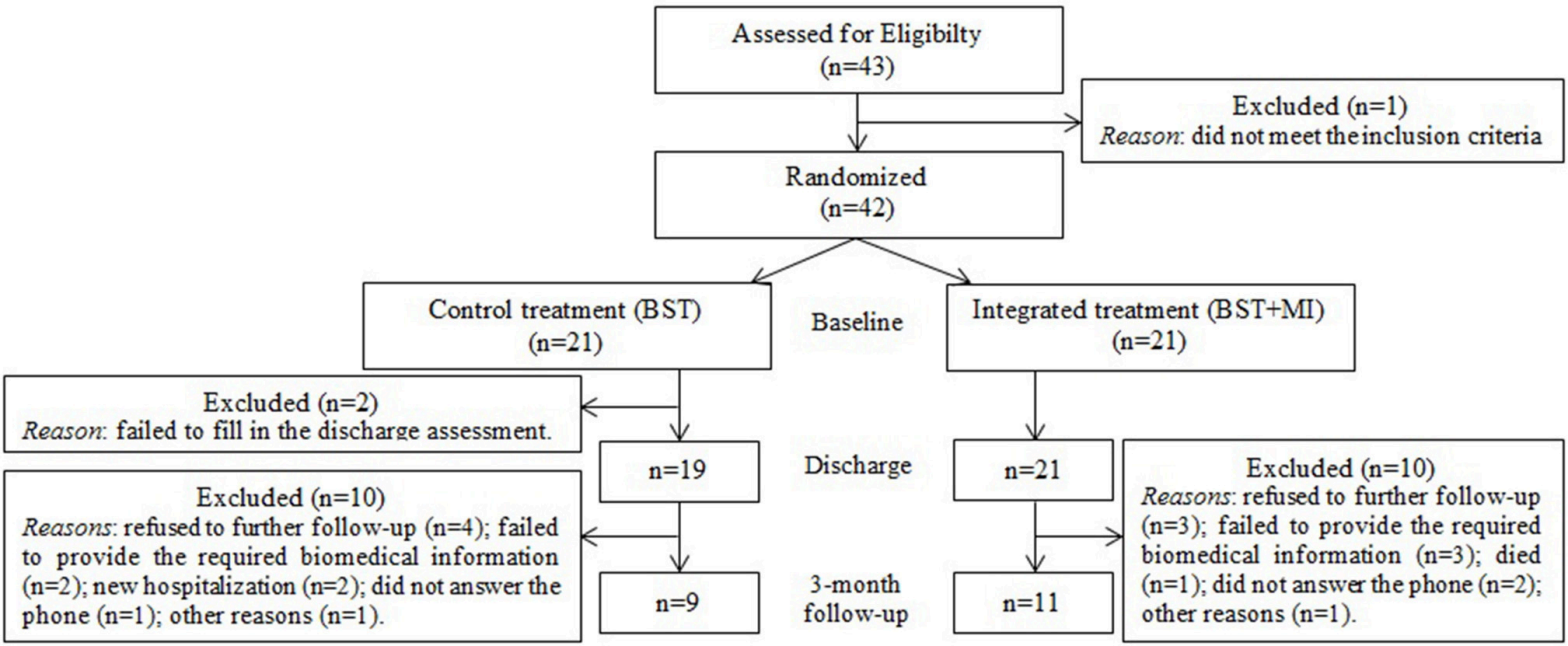
**Study flowchart**.

A total of 42 heart inpatients referred to a single clinical center (Ospedale San Giuseppe, IRCCS Istituto Auxologico Italiano) to attend a 1-month CR and weight-loss treatment were selected by the CR psychotherapist for admission into the study, at the beginning of the rehabilitation program (Figure 1). The following *inclusion criteria* were applied: (1) scoring below 60 on the Psychological General Well Being Index (PGWBI; Grossi et al., 2006); (2) having requested a psychological consultation from the treating cardiologist; (3) being born after 1940; (4) having Italian nationality and (5) presenting with chronic cardiac diseases or having recently undergone heart surgery. *Exclusion criteria* for the study were: (1) presenting cognitive or communication problems; (2) having a vision impairment that would make it challenging to fill in the questionnaires; (3) having an uncorrected hearing impairment that could cause difficulty with the intervention.

### Study design and procedure

The incremental efficacy of the integrated treatment (BST + MI) was assessed in a two-armed, prospective, randomized, single-blind pilot study. After pre-treatment evaluation, participants were randomly assigned into the following two conditions:

1. *Experimental treatment* (BST + MI): standard CR including three sessions of Brief Strategic Therapy combined with Motivational Interviewing principles and techniques.
2. *Control treatment* (BST): standard CR including three individual sessions of Brief Strategic Therapy without provision of Motivational Interviewing.

The randomization scheme was generated by using the method of randomly permuted blocks on the website Randomization.com (www.randomization.com). The number of subjects per block was set to 2 and the number of blocks was set to 21. All the operations were done by an independent Ph.D. student in neuropsychology working in the same hospital where the trial was run. She was the only repository of the randomization plan and matched patients to one of the two conditions sequentially after knowing only their IDs. Patients were blinded about the treatment received.

Sessions took place once weekly in a face-to-face setting and lasted between 30 and 45 min. The same psychotherapist, a specialist in BST and competent in providing MI, delivered both treatments.

Psychological consultations occurred always in the same room. Those subjects assigned to the experimental condition received the BST treatment first; then MI's principles and techniques were provided during in the last 15 min of the therapeutic session. Patients assigned to the control group, instead, received only a 45-min standard treatment (BST)—description of the interventions have been described in detail elsewhere (Pietrabissa et al., 2015b).

Assessment of participants took place in four sections: (1) before recruitment, as part of the CR routine psychological assessment, when the PGWBI was administered to all the patients admitted to the CR program by a professional tester unaware of the study aim and procedure; (2) before assigning patients to the treatment conditions and (3) at discharge from the hospital, by an independent clinical psychologist. Instructions were given before the questionnaires were administered and the professional assisted the participants during compiling; (4) after 3 months, through phone-interviews conducted by trained apprentices in psychology.

Participants did not receive any feedback about their clinical and/or psychological assessment following each time point.

### Psychological outcome measures

Since the Cardiac Rehabilitation European Guidelines extensively encourage the monitoring of cardiac patients' HRQoL, in both the presence and absence of significant psychological distress (Piepoli et al., 2016), the *PGWBI* (Grossi et al., 2006) is routinely administered at the beginning and termination of a CR program. The PGWBI is a 22-item questionnaire that produces a self-perceived evaluation of psychological well-being, expressed by a summary score through six dimensions: Anxiety, Depressed Mood, Positive Well-being, Self-Control, General Health, and Vitality. Its internal consistency ranges from 0.90 to 0.94 (Grossi et al., 2006). The PGWBI can be used alone or in combination with other generic and disease specific questionnaires, both in general populations and in studies of chronic illness, however, the majority of applications have been in studies of cardiovascular disease.

The Italian translation and cultural adaptation of the following self-report questionnaires was also administered in a dedicated room at inclusion and discharge from the hospital.

#### Brief illness perception questionnaire (B-IPQ)

The *Brief Illness Perception Questionnaire (B-IPQ;* Hirani et al., 2006; Giardini et al., 2007), assessing the patients' cognitive and emotional representations of their disease. It traditionally comprises 9 items on a 10-point Likert-scale. For the present investigation, item 9 (“*Please list in rank-order the three most important factors that you believe caused your illness*”) was removed due to obvious difficulties experienced by the participants in answering the question during the preliminary administration of the protocol. The first five items assess the individuals' cognitive representations of the disease; two items (6 and 8) measure the emotional representations of the illness, while item number 7 assesses the patients' degree of understanding of their own illness. Each of the eight items measures an established illness perception dimension (Consequences; Timeline; Personal Control; Treatment Control; Identity; Illness Concern; Coherence; Emotional Representation) and their sum makes up the overall patients' perception of their disease. A high *consequences* score means that the participant see the illness as having major consequences; a high *timeline* score means that he or she thinks the illness will last for a long time; a high *personal control* score means that the participant perceives having good control of the illness; a high *treatment control* score means that the participant believes the treatment is extremely helpful in managing the illness; a high *identity* score indicates that the participant experiences more than one symptom; a high *illness concern* score means that the participant is highly concerned about the illness; a high *coherence* score means that the participant understands the illness and a high *emotional representation* score means that the participant's illness has an extreme effect on his or her emotions (Ng, 2012). In the present sample, the internal consistency for the B-IPQ total score was 0.68.

#### Hospital anxiety and depression scale (HADS)

The *Hospital Anxiety and Depression Scale* (HADS; Annunziata et al., 2011) is composed of 14 items (seven of them relate to anxiety and depression, respectively) to which patients respond on a 4-point Likert scale. The questionnaire was specifically designed to screen for the presence of emotional disorders among patients with organic diseases in clinical settings. It is short and rapid in administration (taking 2–5 min) and generally well-accepted (Mykletun et al., 2001). Among the study participants, the HADS Cronbach's α coefficient was 0.88 (0.82 for anxiety and 0.80 for depression).

#### Barratt impulsiveness scale (BIS-11)

The *Barratt Impulsiveness Scale* (BIS-11; Fossati et al., 2001) is a 30-item questionnaire assessing the presence of impulsive behaviors/traits on a 4-point Likert scale. Since impulsiveness is a multi-faceted construct, a total score, as well as scores for three second-order factors (*attentional impulsiveness*—or lack of cognitive persistence with an inability to tolerate complexity; *motor impulsiveness*—or acting on the spur of the moment; and *no-planning impulsiveness*—or lack of a sense of the future) and six first-order factors (*attention, motor, self-control, cognitive complexity, perseverance*, and *cognitive instability*) are measured (Patton et al., 1995). In the present sample, the BIS-11 Cronbach's α coefficient was 0.71 (0.55 for *attentional impulsiveness*; 0.65 for *motor impulsiveness*; 0.73 for *no-planning impulsiveness*).

#### General self-efficacy scale (GSE or GSES)

The *General Self-Efficacy Scale* (GSE or GSES; Zotti et al., 2007) is a 10-item psychometric scale used to evaluate the individual's perceived self-efficacy regarding coping and adaptation abilities in both daily activities and isolated stressful events. It specifically measures persons' *optimistic* self-beliefs to cope with a variety of difficult demands in life. All items are classified on a 4-point scale assessing the degree of the respondent's agreement to the proposed statements. The internal consistency for the GSE total score among the present sample was 0.85.

#### 12-Item Short Form Health Survey (SF-12)

The *12-Item Short Form Health Survey* (SF-12; Lim and Fisher, 1999; Jakobsson, 2007) is a shorter alternative to the 36-item Short Form Health Survey (SF-36) frequently used in health outcomes surveys to measure Health Related Quality of Life (HRQoL; Ware et al., 1996; Gandek et al., 1998). The questionnaire relies on eight subscales (physical functioning—PF, 2 items; role limitations due to physical problems—RP, 2 items; bodily pain—BP, 1 item; general health perceptions—GH, 1 item; vitality—VT, 1 item; social functioning—SF, 1 item; role limitations due to emotional problems—RE, 2 items; and mental health - MH, 2 items). Results produce two synthetic indices, respectively related to the individuals' physical and mental state: the *Physical Component Summary* (PCS) of the first four domains, and the *Mental Component Summary* (MCS) composed of the last four. The psychometric properties and factor structure of the SF-12 have been examined in several studies worldwide, showing this scale to be a reliable and valid measure for a variety of population groups (Jenkinson and Layte, 1997; Lim and Fisher, 1999; Delate and Coons, 2000; Gandhi et al., 2001; Jayasinghe et al., 2009; Montazeri et al., 2011), and specifically for a person with a heart illness. The PCS and MCS Cronbach's α coefficients in the present sample were 0.75 and 0.80, respectively.

#### Readiness-to-Change ruler (RR)

The *Readiness-to-Change ruler* (RR) is used as a quick assessment of a person's motivation to change a specific health-related behavior and serves as the basis for motivation-based interventions aimed at eliciting behavioral change. The ruler describes a continuum from “not prepared to change” on the left, to “already changing” on the right, on a scale from 1 to 10. Assessing readiness to change helps clarify the patients' *willingness* to modify unhealthy lifestyles, to evaluate how *important* this purpose is for them, and to assess their *confidence* to succeed. Low scores range from 0 to 3; scores between 4 and 6 indicate uncertainty; 7 to 8 and high scores of 9 to 10 are good predictors of change.

#### Treatment self-regulation questionnaire (TSRQ)

The *Treatment Self-Regulation Questionnaire* (TSRQ; Levesque et al., 2007) evaluates the degree to which a person's motivation to engage in a specific healthy behavior is self-determined. Specifically, persons are asked to indicate the extent to which each reason for engaging in a specific health behavior is true for them, using a 7-point scale that goes from “not at all true” to “very true.” The scale is based on the SDT (Deci et al., 1994; Deci and Ryan, 2012) and, for the purpose of this study, comprised 13 items clustered into four dimensions: *external regulation* (items 2, 9, and 11), *introjected regulation* (items 1, 4, 6, and 10), *identified regulation* (items 5, 8, and 13) and *intrinsic regulation* (Items 3, 7, and 12). Subscale scores can be used separately, or a RAI can be calculated. In the present sample, the *external regulation* Cronbach's α coefficient was 0.78, the Cronbach's α coefficient for *introjected regulation* was 0.75, and the internal consistency for *identified regulation* and *intrinsic regulation* were 0.82 and 0.74 respectively.

Demographic information (age, gender, marital status, employment status, education) was collected by self-report at pre-treatment administration. On that occasion, patients were also asked to sign the written and informed consent to take part in the study.

Patients who did not provide the informed consent were excluded from the trial (*n* = 0).

### Biomedical outcome measures

As part of the routine outcome assessment of CR programs, study participants' (a) *Kilograms* (Kg), (b) *Body Mass Index* (BMI), (c) *Systolic Blood Pressure* (SBP), (d) *Low-Density Lipoprotein Cholesterol* (LDL), and (e) *Glycaemia* were collected from their medical record at inclusion and discharge from the hospital. The presence of *diabetes* and the individuals' *smoking status* were also registered at inclusion to the cardiac unit.

### Follow-up assessment

Three months after discharge, four questionnaires were re-administered to each participant: the *General Self-Efficacy Scale*, the *12-Item Short Form Health Survey*, the *Readiness-to-change Ruler* and the *Treatment Self-Regulation Questionnaire* through phone-interviews. Individuals' *weight, SBP, LDL*, and *Glycaemia* were also collected by self-report.

Trained apprentices in psychology had contacted participants a week before to inform them that they would receive another phone call in 7 days (that is 3 months after discharge) and asked them to monitor and register these parameters during that time (at home or at a pharmacy).

### Treatment fidelity

The quality of the application of MI's principles and techniques were assessed by a specialist in the field. MI sessions were audio recorded and transcribed verbatim; a random sample of them were critically analyzed (Moyers et al., 2016).

### Sample size calculation

Given that MI integrated with BST has never been applied to cardiovascular patients before, the MOTIV-HEART trial is similar to a pilot study, defined as a small-scale research study carried out in preparation for larger investigations. According to Lackey and Wingate (Lackey and Wingate, 1986), a pilot study may use a minimum of 10% of the sample required for a standard study. Given that 428 participants (214 per group) are needed for a Student's *t*-test to detect a medium standardized difference (Cohen's d equal to 0.35) with a statistical power of 0.95 and alpha set to 0.05 (two-sided; G^*^Power 3.1.2 software), 42 subjects were deemed sufficient for the present investigation.

### Statistical analysis

Data were analyzed according to the intention-to-treat approach and by means of exact non-parametric tests due to non-normality of several distributions In particular, the chi-square test and the Spearman rho were used to assess the bivariate associations between variables, while the Mann-Whitney U-test and the Wilcoxon signed rank test were used to assess the statistical significance of between-group and within-group differences, respectively. Critical alpha was set to 0.05 and the Monte Carlo method was applied to the calculation of all *p*-values. All analyses were run using the Statistical Package for the Social Sciences (SPSS) software 20.0 for Windows (SPSS, version 20.0; SPSS, Inc., Chicago, IL).

## Results

### Baseline characteristics

Forty-two patients (25 males and 17 females) were included in the trial and were equally distributed into the two conditions (*n* = 21). The overall mean age was 60.49 (*SD* = 8.22) and the overall mean BMI was 42.03 (*SD* = 16.12) (see Table 1). Nearly half (47.6%) of the study participants were retired, while 33.3% were employed. Regarding their marital status, 57.1% of the sample were married and 23.8% were separated or divorced. Also, 50% of respondents had a high school education level. Twenty out of fourty-one patients (48.8%) received a diagnosis of diabetes while, with regard to their smoking habits, the overall smoking rate was 26.8, and 41.5% of the study participants fell into the ex-smoker category.

**Table 1:** **Baseline characteristics and biomedical parameters of the sample**.

**Observations**	***N***	**BST (*****n*** = **21)**		**BST** + **MI (*****n*** = **21)**		**Statistics**^a^	
	***n*%**	***N***	**%**	***N***	**%**	**Chi2**	***p***
**GENDER**							
Male		9	42.9	16	76.3	6.222	0.028^*^
Female		12	57.1	5	23.7		
**EMPLOYMENT STATUS**							
Worker		4	19.0	10	47.6	3.905	0.299
Housewife		3	14.3	2	9.5		
Unemployed		2	9.6	1	4.8		
Retired		12	57.1	8	38.1		
**EDUCATION**							
Junior school		4	19.0	1	4.8	7.914	0.043^*^
Middle school		4	19.0	9	42.8		
High school		13	62.0	8	38.1		
University		−	−1	3	14.3		
**MARITAL STATUS**							
Single		−	−	2	9.5	4.833	0.203
Married		11	52.4	13	61.9		
Separated/Divorced		5	23.8	5	23.8		
Widowed		5	23.8	1	4.8		
**OBESITY**							
No		2	10.5	2	9.5	0.011	1.000
Yes		17	89.5	19	90.5		
**SMOKER**							
No		7	35.0	6	28.6	0.930	0.671
Yes		4	20.0	7	33.3		
Ex		9	45.0	8	38.1		
**DIABETES**							
No		11	55.0	10	47.6	0.223	0.758
Yes		9	45.0	11	52.4		
		Median	IQI^b^	Median	IQI	Mann-Whitney U	*p*
Age		63.6	55.3–68.8	61.6	54.2–65.3	189.0	0.440

a *Monte Carlo p estimation*.

b *InterQuartile Intervals*.

* *p*.

With the exception of two patients who did not complete the questionnaires at discharge, 42 participants completed both the pre- and post-treatment assessments, and received the experimental (*n* = 21) or the usual (*n* = 21) treatment. The majority of the subjects had a specific intention to change their eating habits (*n* = 26), 12 respondents to exercise more, while four subjects said their main motivation was to stop smoking.

### Post-treatment between-group comparisons

The analysis of the data collected at discharge from the CR program did not show any significant between-group difference in the outcome variables (Table 2).

**Table 2:** **Pre-post treatment data for the two groups**.

**Variable**		**Median and IQI**	**BST** ***n*** **PRE** = **21** ***n*** **POST** = **19**		**BST** + **MI (*****n*** = **21)**		**Statistics**	
			**Median**	**IQI**	**Median**	**IQI**	***U*^b^**	***P*^a^**
LDL (mg/dL) Optimal: ≤100^*^		Pre	99.0	83.0–127.0	96.0	75.5-149.5	194.5	0.895
		Post	76.0	61.0–108.0	67.0	60.5-95.5	167.5	0.398
Glycaemia (mg/dL) Optimal: ≤100		Pre	114.0	90.0–175.0	116.0	99.0–194.5	186.5	0.732
		Post	102.0	93.0–120.0	99.0	87.5–118.0	164.5	0.353
SBP (mmHg) Optimal: <140		Pre	117.0	113.0–123.0	123.0	117.5–130	119.5	0.036^*^
		Post	117.0	105.0–127.0	117.0	102.5–130.0	193.5	0.878
Weight (Kg)		Pre	98.1	92.0–106.2	119.9	99.7–128.4	100.5	0.007^*^
		Post	95.0	88.8–102.7	116.2	98.7–124.3	95.5	0.005^*^
BMI (kg/m^2^) Obesity class I: 30.0–34.9 Obesity class II: 35.0–39.9 Obesity class III: ≥40.0		Pre	38.1	36.0– 46.1	40.3	35.98–44.4	181.5	0.643
		Post	36.9	34.5–44.2	39.3	35.6–42.6	179.5	0.604
B-IPQ	Consequences	Pre	7.0	5.0–8.0	7.0	5.0–8.0	212.0	0.836
		Post	6.0	5.0–7.0	7.0	5.0–8.0	174.0	0.491
	Timeline	Pre	10.0	6.5–10.0	10.0	7.5–10.0	195.5	0.501
		Post	10.0	6.0–10.0	10.0	6.0–10.0	196.0	0.924
	Personal control	Pre	4.0	2.0–6.0	5.0	3.0–8.5	178.0	0.280
		Post	4.0	3.0–6.0	4.0	3.0–6.5	178.0	0.572
	Treatment control	Pre	2.0	1.0–3.0	3.0	1.5–4.5	137.0	0.032^*^
		Post	2.0	1.0–3.0	2.0	1.0–3.0	193.5	0.876
	Identity	Pre	6.0	4.0–7.0	7.0	3.5–8.0	191.0	0.465
		Post	4.0	2.0–7.0	6.0	3.0–7.0	147.0	0.153
	Illness Concern	Pre	7.0	5.5–8.5	7.0	5.0–8.0	210.5	0.804
		Post	7.0	3.0–8.0	6.0	3.5–7.5	184.0	0.675
	Coherence	Pre	4.0	2.0–6.0	3.0	1.5–5.0	185.0	0.378
		Post	3.0	2.0–4.0	3.0	1.0–5.0	189.0	0.783
	Emotional representation	Pre	5.0	4.5–8.0	5.0	5.0–8.0	216.5	0.924
		Post	5.0	4.0–7.0	6.0	5.0–7.5	161.0	0.290
HADS	Anxiety	Pre	10.0	5.5–14.5	9.0	8.5–11.0	213.5	0.872
		Post	6.0	2.0–10.0	6.0	5.5–9.0	172.5	0.452
	Depression	Pre	7.0	5.0–12.0	8.0	5.5–11.0	200.0	0.613
		Post	7.0	5.0–9.0	6.0	3.5–9.0	198.5	0.985
	Total score	Pre	16.0	11.5–26.0	18.0	14.0–21.5	210.0	0.799
		Post	14.0	9.0–16.0	13.0	10.0–16.5	195.0	0.867
BIS-11	Motor impulsiveness	Pre	22.0	19.0–26.0	22.0	19.5–27.0	201.0	0.638
		Post	21.0	17.0–27.0	22.0	19.5–25.5	193.5	0.881
GSE	Total score	Pre	2.8	2.4–3.1	2.9	2.5–3.1	206.5	0.741
		Post	2.9	2.7–3.5	2.9	2.6–3.5	183.5	0.862
SF-12	PCS	Pre	34.9	28.1–38.0	30.3	25.3–39.7	194.0	0.518
		Post	36.7	34.4–45.2	35.8	27.0–42.7	159.0	0.287
	MCS	Pre	36.1	27.7–45.1	40.6	35.0–49.7	157.0	0.118
		Post	50.8	40.4–56.6	53.0	45.6–55.1	176.0	0.555
RR	Willingness	Pre	8.0	6.5–9.0	10.0	7.0–10.0	134.0	0.028^*^
		Post	8.0	8.0–10.0	8.0	7.5–10.0	196.0	0.921
	Importance	Pre	9.0	8.0–10.0	10.0	8.5–10.0	172.0	0.187
		Post	10.0	8.0–10.0	10.0	9.0–10.0	192.5	0.844
	Confidence	Pre	8.0	5.0–8.5	7.0	6.5–8.0	212.5	0.847
		Post	8.0	7.0–9.0	8.0	6.0–9.0	198.5	0.985
TSRQ	External regulation	Pre	3.7	2.2–5.5	3.0	1.0–5.0	177.5	0.276
		Post	2.0	1.0–4.7	3.0	1.5–4.0	157.5	0.258
	Introjected regulation	Pre	5.2	4.1–6.2	4.5	2.7–6.2	176.0	0.271
		Post	4.5	3.7–5.5	4.5	3.6–5.2	190.0	0.810
	Identified regulation	Pre	7.0	5.8–7.0	7.0	6.7–7.0	197.5	0.355
		Post	7.0	6.0–7.0	7.0	6.3–7.0	195.0	0.904
	Intrinsic regulation	Pre	6.0	5.2–7.0	6.0	4.0–7.0	192.5	0.493
		Post	6.0	5.3–7.0	6.0	5.0–6.8	183.5	0.670
	RAI	Pre	3.8	1.2–10.4	7.3	2.6–9.2	190.0	0.464
		Post	9.7	3.4–13.8	8.4	2.3–12.2	160.5	0.307

*B-IPQ, Brief Illness Perception Questionnaire; HADS, Hospital Anxiety and Depression Scale; BIS-11, Barratt Impulsiveness Scale; GSE, General Self-Efficacy Scale; SF-12, 12-Item Short Form Health Survey; RR, Readiness-to-Change ruler; TSRQ, Treatment Self-Regulation Questionnaire; Relative Autonomous Motivation Index; PCS, Physical Component Summary; MCS, Mental Component Summary; Kg, Kilograms; BMI, Body Mass Index; SBP, Systolic Blood Pressure; LDL, Low-Density Lipoprotein Cholesterol; IQI, InterQuartile Intervals*.

* *Optimal (for patients with diabetes and/or Coronary Heart Disease, CHD): <70*.

a *Monte Carlo method*.

b *Mann-Whitney U-test*.

* *p*.

### Within-group changes

Even if no significant between-group difference was detected between the two conditions at the end of the CR program, the analysis of pre-post changes revealed that *external regulation* decreased significantly only in the BST group. However, an inspection of the interquartile ranges showed that patients allocated to the BST condition had higher scores in that measure at baseline in comparison to patients assigned to the experimental group. Also the *RAI* and the *willingness to change* increased significantly only in the BST group but, similarly to *external regulation*, the interquartile ranges of baseline scores were lower in the BST than in the experimental group.

### Within-group changes at the 3-month follow-up

A number of participants (BST = 12; BST + MI = 10) dropped out of the study and were lost to follow-up. This prevented a reliable evaluation of between-group differences and allowed only the more powerful analysis of within-group changes. In comparison to the end-of-treatment data, results showed a significant improvement in both conditions only in the SBP. No other variables changed to a statistically significant extent.

## Discussion

According to the results of the present investigation, the addition of motivational principles and techniques to the standard psychological treatment (BST) did not contribute to the average improvement of either biomedical or psychological outcomes among CR patients.

Few significant within-group changes at discharge were observed for the TSRQ-*external regulation* dimension, the TSRQ-*Relative Autonomous Motivation Index (RAI)* and the inpatients' *willingness to change*, which significantly improved only in the BST condition. The inspection of baseline data revealed that patients assigned to that condition had a higher median and higher interquartile ranges in the TSRQ-*external regulation* dimension than the BST + MI condition. Similarly, those receiving the BST + MI treatment had a higher median and higher interquartile ranges in both the *RAI* and *willingness to change*. These differences are probably due to a randomization flaw and also to defects in the selection criteria. In fact, albeit the patients' readiness to change represented one of the key-outcome variables of the present study and was measured before treatment, participants were neither selected nor randomized on its basis. Likewise, several other factors were not controlled, such as age and gender of the participants. While the BST + MI condition contained a majority of men, the other condition was mainly composed of women, which could have biased the treatment effects on both biomedical (Kg and SBP) and psychological outcomes (*B-IPQ-treatment control* dimension and *willingness to change*). Another limitation of the study is the low reliability of several measures. In fact, with the exception of the HADS, the MCS of the SF-12, and the TSRQ*-identified regulation* dimension, the measures all showed an unsatisfactory internal consistency. Moreover, the residential setting might have played a role in influencing the interventions' outcomes. In fact, besides affecting the research procedure from a methodological point of view, CR, by its own nature, has a motivating effect on the subjects. Firstly, the physical, psychological and social effects of participating in CR activities may themselves lead to reconstruction of patients' attitudes and beliefs about the likelihood of overcoming any difficulties in maintaining health-related behaviors (Berkhuysen et al., 1999). Taking part in group-based exercise also helps patients to gain a common identity arising from the interaction with other persons sharing similar needs, conditions and purposes, thus further encouraging their participation in the CR program, decreasing their fears and concerns, and promoting self-confidence in goal achievements (Clark et al., 2005; Midtgaard et al., 2006). Finally, the caring behavior of rehabilitation team members acts as another important motivational factor that positively encourages the participants' involvement in the CR program.

Learning about a healthier life style, recognizing their own weakness and ability, and the recovery of physical capability by performing activities are effective results of participating in CR that affect the psychosocial aspect of patients' lives and increase their overall QoL (Williams et al., 2006). Inadequate social support and perceived or real social isolation would, instead, likely make the individuals more aware of their daily hassles and life stressors, thus significantly compromising their allegiance to CR programs over the long term (Heo et al., 2014). In fact, attrition rates for CR programs are still critical worldwide (Scotto et al., 2011). In this study, half of participants did not respond to the 3-month follow-up call and this prevented the valid and reliable evaluation of outcomes after the residential period.

While maximizing psychological health is a core goal of CR, psychological care can be fragmented and patchy. Nearly all of the suggested theories and models about patients' adherence to treatment regimens recognize that enhancing their intrinsic motivation to actively participate is an important way to ensure adherence to the CR program (Richards et al., 2016). Still, the role of psychosocial components cannot be excluded or isolated (Ladwig et al., 2014). Several controlled studies have investigated the effectiveness of psychotherapeutic interventions such as Cognitive Behavioral Therapy (CBT; Barth et al., 2005; Davidson et al., 2010), Interpersonal Therapy (IT; Lespérance et al., 2007), and “collaborative treatment” (Katon et al., 2010) in addressing psychosocial issues in CVD patients. Positive, yet moderate effects were reported in most of the studies, but no differences were detected in the main outcomes between the study groups. However, methodological issues that were raised suggest that research findings do not prove the effectiveness of the interventions (Ladwig et al., 2014). Therefore, although it is possible that individualized approaches may be more likely to improve prognosis, the most suitable form of psychological treatment in CR has yet to be determined.

The present pilot study is unique in investigating the effect of BST, as well as the incremental efficacy resulting from the combination of BST with MI over and beyond the stand-alone BST, in an in-patient and telephone-based outpatient CR program.

Treatment leakage cannot be excluded: it may have been difficult, in fact, for the same psychotherapist to deliver both interventions and ensure MI techniques of sufficient quality.

Still, besides their use to evaluate the preliminary effects of an intervention, pilot studies are particularly valuable for recognizing inconsistencies and misapplication of research designs and procedures, leading to misinterpretations of study outcomes. Generalizability of the trial findings are therefore limited. A stronger experimental design that can overcome the limitations highlighted above, and that includes a higher number of participants, would surely help researchers and clinicians to clarify the real impact of both BST and the integrated treatment on clinical outcomes among persons with CVD.

## Conclusions

Achieving a reduction in modifiable risk factors and improving adherence to treatment are primary goals of CR and secondary prevention programs, and psychological variables play an important role in determining the long-term maintenance of the results attained during the CR program. Psycho-educational support can facilitate behavioral change by providing patients with information about the disease and associated risk factors, helping them to cope with emotional problems, and fostering positive attitudes toward change (Child et al., 2010) However, increasing individuals' knowledge does not necessarily lead to effective behavioral change (Dunn et al., 1990). Too often, in fact, persons are aware of their need to alter an unhealthy lifestyle but, for several reasons (e.g., lack of motivation or self-efficacy, perception that excessive effort is required, previous relapses, lack of social support, etc.), they are caught up in their own ambivalence.

By increasing the patients' health status, and helping them to perceive that achievements are the result of their own efforts, CR can have a strong motivating effect. With the termination of the rehabilitation program, however, the patients' monitoring and support provided by health care professionals also comes to an end, and if patients still doubt their own abilities to cope with daily-life difficulties, relapses are more likely to occur (Shahsavari et al., 2012).

Indeed, deciding to undertake a CR program does not necessarily equate to being intrinsically motivated to change, and the rehabilitation program is often seen by people as a way to avoid responsibility for taking autonomous action to improve their health.

Effective self-management is crucial for patients in order to achieve long-term positive outcomes, and adherence to treatment recommendations has been reported as particularly low among heart patients (Cooper et al., 1999).

MI is a counseling style that marries well with the principles of CR in terms of increasing self-efficacy and readiness to change among heart patients, promoting a client-centered approach, and encouraging cost- and time-effective treatment procedures (Castelnuovo et al., 2014). Although this pilot study does not provide evidence of the benefit of adding MI to the standard CR psychological treatment, it surely helps in understanding barriers to and facilitators of implementation of the intervention, as well as offering empirical evidence of study parameters. Therefore, positive effects in CR and cardiovascular secondary prevention cannot be excluded based on the results of the present investigation, and both the integration of MI with BST and the delivery of BST interventions within CR are still worthy of further examination. In fact, a number of patients enrolled in the study showed high motivation to change at baseline, and some evidence revealed that applying MI to people who are already ready to change may actually slow down their progress (Stotts et al., 2001; Rohsenow et al., 2004).

Moreover, self-report instruments must be selected carefully from the growing literature on motivation and lifestyle change to fit the uniqueness of the patients and of the clinical setting. Their validity and reliability must be addressed, as outcome measures need to be valid and sensitive to longitudinal and between-group differences in order to stimulate comparison of outcomes between studies.

## Ethics statement

The study was approved by the Ethics Committee of the Istituto Auxologico Italiano. All procedures were run in accordance with the 1964 Helsinki declaration and its later amendments.

## Author contributions

GP and GM conceived the study, analyzed and interpreted the data and drafted the manuscript. AR contributed to the analysis and interpretations of the data and drafted the manuscript. GC revised the manuscript critically for important intellectual content. All authors approved the version to be published and agree to be accountable for all aspects of the work in ensuring that questions related to the accuracy or integrity of any part of the work are appropriately investigated and resolved.

### Conflict of interest statement

The authors declare that the research was conducted in the absence of any commercial or financial relationships that could be construed as a potential conflict of interest.
